# HSV-1 interaction to 3-*O*-sulfated heparan sulfate in mouse-derived DRG explant and profiles of inflammatory markers during virus infection

**DOI:** 10.1007/s13365-017-0521-4

**Published:** 2017-03-21

**Authors:** Harsh Sharthiya, Chanmoly Seng, T. H Van Kuppevelt, Vaibhav Tiwari, Michele Fornaro

**Affiliations:** 1 Department of Anatomy, Chicago College of Osteopathic Medicine, Midwestern University, Downers Grove, IL 60515 USA; 2 Department of Biomedical sciences, College of Health Sciences, Midwestern University, Downers Grove, IL 60515 USA; 3 Department of Biochemistry, Nijmegen Institute for Molecular Life Sciences, Radboud University, 6500 HB Nijmegen, The Netherlands; 4 Department of Microbiology and Immunology, Chicago College of Osteopathic Medicine, Midwestern University, Downers Grove, IL 60515 USA

**Keywords:** HSV entry, Heparan sulfate, Sensory neurons, Neuroinflammation

## Abstract

The molecular mechanism of herpes simplex virus (HSV) entry and the associated inflammatory response in the nervous system remain poorly understood. Using mouse-derived ex vivo dorsal root ganglia (DRG) explant model and single cell neurons (SCNs), in this study, we provided a visual evidence for the expression of heparan sulfate (HS) and 3-*O*-sulfated heparan sulfate (3-*O*S HS) followed by their interactions with HSV-1 glycoprotein B (gB) and glycoprotein D (gD) during cell entry. Upon heparanase treatment of DRG-derived SCN, a significant inhibition of HSV-1 entry was observed suggesting the involvement of HS role during viral entry. Finally, a cytokine array profile generated during HSV-1 infection in DRG explant indicated an enhanced expression of chemokines (LIX, TIMP-2, and M-CSF)—known regulators of HS. Taken together, these results highlight the significance of HS during HSV-1 entry in DRG explant. Further investigation is needed to understand which isoforms of 3-*O*-sulfotransferase (3-*O*ST)-generated HS contributed during HSV-1 infection and associated cell damage.

**Electronic supplementary material:**

The online version of this article (doi:10.1007/s13365-017-0521-4) contains supplementary material, which is available to authorized users.

## Introduction

The hallmark of herpes simplex virus type 1 (HSV-1) infection is to establish life-long latency in the sensory ganglia mainly in the dorsal root ganglia (DRG) of the host following an initial infection in epithelial cells (Roizman and Whitley 2013; Fraser and Valyi-Nagy 1993; Wilson and Mohr 2012). Upon reactivation, the virus resumes its cycle of lytic gene expression to replicate successfully and transport newly made virions axonally back either to peripheral nervous system, resulting in cold sores or fever blisters and corneal keratitis, or central nervous system resulting in meningitis and encephalitis in the brain (Whitley and Roizman 2001; Nicoll et al. 2012; Simmons 2002).

The process of HSV-1 entry initiates with the specific interaction between viral envelope glycoproteins and host cell surface receptors (Antoine et al. 2013; Spear 2004; Hadigal and Shukla 2013; Connolly et al. 2011). The HSV glycoproteins B and C (gB and gC, respectively) mediate their initial attachment to host cell surface heparan sulfate proteoglycans (HSPG) (Shukla and Spear 2001; Tiwari et al. 2015; WuDunn and Spear 1989; Herold et al. 1991; Shieh et al. 1992). The initial binding step of the virus to HSPG results in a conformational change that brings major viral glycoprotein D (gD) binding domain to interact with any given host cell receptors (nectin, HVEM, and 3-*O*-sulfated heparan sulfate; 3-*O*S HS). The later process involves three additional HSV glycoproteins, gB, gH, and gL, and possibly an additional gH co-receptor, which trigger the fusion of the viral envelope with the plasma membrane of host cells (Geraghty et al. 1998; Montgomery et al. 1996; Shukla et al. 1999; Eisenberg et al. 2012).

Interestingly, recent studies widely documented the role of heparan sulfate (HS) and the HS-degrading enzyme heparanase in the pathological process including neuroinflammation (Zhang et al. 2014; Li and Vlodavsky 2009; Parish 2006; Hadigal et al. 2015). Therefore, understanding the involvement of HS and modified HS in HSV-1 entry, spread, and cell damage at the molecular level will aid in the development of novel strategies to prevent virus spread and associated inflammation (Joyce et al. 2005; Rusnati et al. 2005; Ferro et al. 2007; Tiwari et al. 2011; Raman et al. 2012). In this study, we investigated the interaction between HSV-1 glycoproteins and HS (Yabe and Maeda 2015) and modified 3-*O*S HS generated by the enzyme 3-*O*-sulfotransferase (3-*O*ST) (Yabe et al. 2005).

In order to achieve our goal, we developed a mouse-derived ex vivo DRG explant and single cell neuronal (SCN) cultures as a model to study HSV-1 entry. We observed the susceptibility and replication of KOS HSV-1 strain in the DRG explants. In addition, our data further provided the visual and quantitative evidence to support the significance of HS, 3-*O*S HS, in promoting HSV entry and inducing changes in the pattern of expression of unique sub-set of chemokines. Finally, we propose that DRG-based ex vivo model provides a unique platform to further investigate which form of HS is critical for viral entry and cell damage.

## Material and methods

**Fig. 1:**
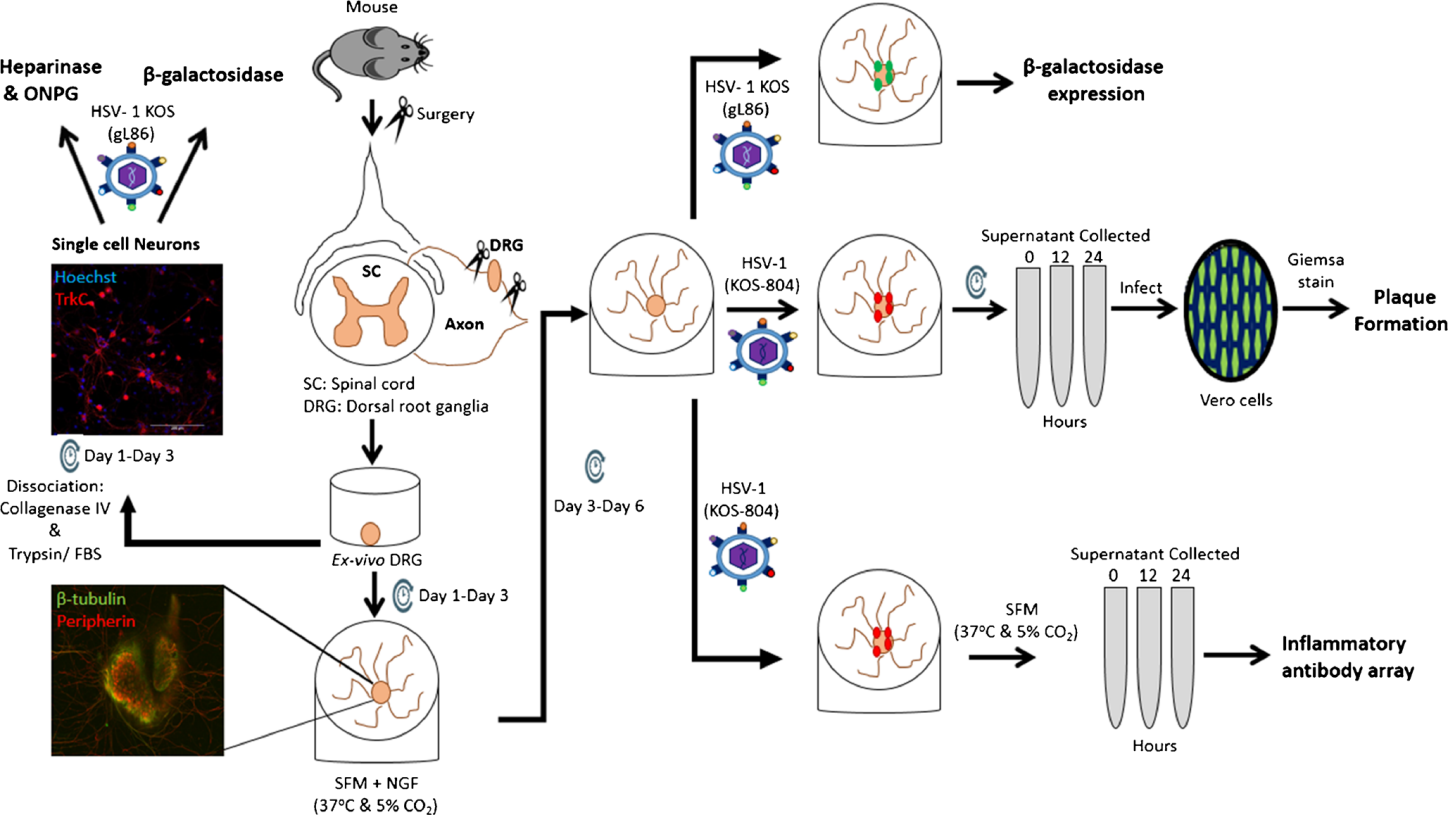
Schematic representation of the experimental procedures carried out in this study. DRGs were isolated from adult NIH/Swiss mice and either plated ex vivo as a whole explant or dissociated into single cell cultures composed of neurons and satellite cells. These two models were infected with HSV-1 KOS (gL86) reporter virus for the β-galactosidase and heparinase/ONPG assay. HSV-1 (KOS-804) replication competent virus was used for plaque formation and inflammatory antibody array

An overall experimental design is schematically described in the Fig. 1.

### Harvesting of DRG

In accordance with institutional review board-approved protocols (IACUC—Midwestern University), mouse-derived DRG explants were isolated from adult NIH/Swiss mice (Harlan Laboratories, Chicago, IL). Briefly, the animals were euthanized and the vertebral column was surgically dissected. The vertebral canal was exposed by performing a double cut on both sides of the vertebral bodies using fine scissors. The ventral access through the vertebral bodies did not interfere with the DRG located along the dorsal roots. DRGs were localized on both sides of the spinal cord, removed, and cleaned from excess of fibers and connective tissue. DRG explants were then plated ex vivo in 12-well plates pre-coated with matrigel (Sigma-Aldrich, St. Louis, MO) in serum-free medium (SFM) (Fueshko and Wray 1994) and maintained in culture conditions (37 °C and 5% CO_2_) for up to 6 days. The medium was changed every 72 h.

### Generating single cell neurons from DRGs

After collection, explants were immediately placed in F12 media (Gibco, Waltham, MA) containing collagenase (1.25 mg/ml) (Sigma-Aldrich, St. Louis, MO) and incubated (37 °C and 5% CO_2_) for 45 min. The collagenase step was repeated for another 45 min. Afterward, explants were incubated in F12 media containing trypsin (0.025%, Sigma-Aldrich, St. Louis, MO) mixture for 30 min followed by F12 media-containing FBS (Sigma-Aldrich, St. Louis, MO) for 15 min. Thereafter, explants were washed three times with F12 media and mechanically dissociated with a glass pipette until the solution turned cloudy indicating a successful dissociation. Dissociated cells were filtered through a 0.22-μm filter (BD Falcon, Franklin Lakes, NJ) and centrifuged (2400 rpm) for 2 min. After removing the supernatant, the cells were resuspended in neurobasal media (Sigma-Aldrich, St. Louis, MO) containing B-27 supplement (Life Technologies, Carlsbad, CA), PSN antibiotics (Gibco, Waltham, MA), 0.5 mM l-glutamine (Sigma-Aldrich, St. Louis, MO) and NGF (5 ng/ml, Alomone Labs, Jerusalem, Israel). The resuspended cells were plated on laminin-coated cover slides (Neuvitro, Vancouver, WA).

### HSV-1 infection in DRG explants

Groups of three DRG explants/tubes were infected with HSV-1 gL86 (10,000 virions) in serum-free media. After 12 h of infection at 37 °C, explants were washed with PBS, fixed, and permeabilized, using buffer followed by incubation with X-Gal (5-bromo-4-chloro-3-indolyl-β-d-galactosidase; Invitrogen) at 1.0 mg/ml, which yields an insoluble blue-stained product upon hydrolysis by β-galactosidase. Blue cells (representing viral entry) were seen at multiple locations per explant as shown in the supplementary figures (Fig. 1S). Microscopy was performed using Zeiss Axiovert 100.

### Plaque formation using infected DRGs

**Fig. 2:**
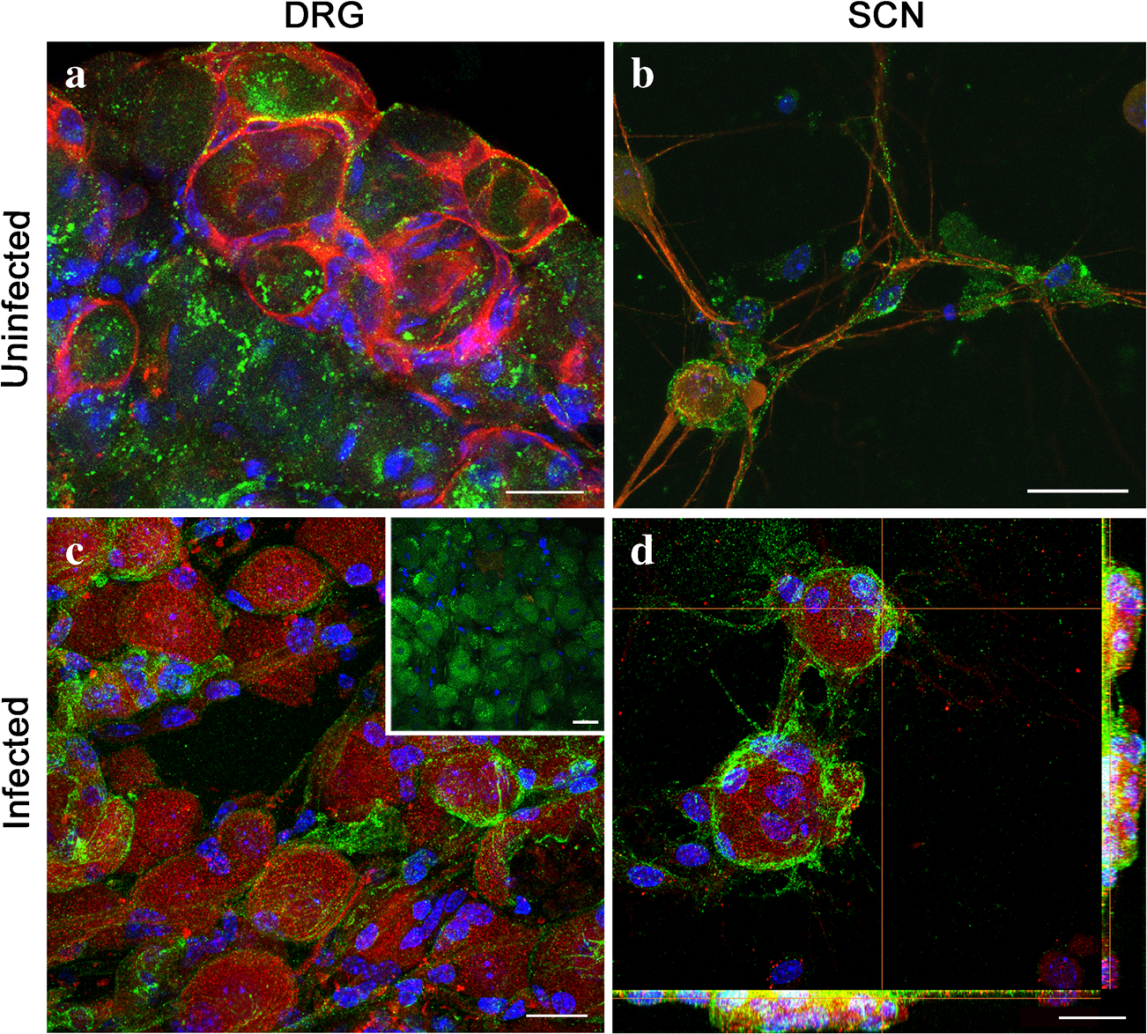
Expression and localization of heparan sulfate (HS) in dorsal root ganglia (DRG) and single cell neurons (SCN). Sections of DRGs (**a**) and SCNs (**b**) were double labeled using the anti-HS 10E4 epitope FITC-conjugated antibody and the specific neuronal marker against TrkC receptor showing that HS is widely expressed on the surface of DRG neurons. Samples were mounted on coverslips with mounting medium containing blue DAPI, which labels the nucleus. HSV-1-infected DRGs and SCNs (**c**, **d**) were then double labeled with anti-glycoprotein B (gB, *red*) and anti-HS marker (*green*). As control, a section of uninfected DRG double labeled with anti-gB and anti-HS antibodies is shown in **c**, *inset*. Confocal imaging displayed sequestration and accumulation of HS (*yellow*) in the membrane region. *Scale bars:* **a**–**d** = 25 μm (color figure online)

A group of three DRG explants per well in triplicate experiment was cultured for 3 days prior to be infected with replication-competent syn mutant HSV-1(KOS-804) (Little and Schaffer 1981) or mock infected with PBS alone as a control for 2 h at 37 °C (Fig. 2S). After removal of the unbound virus, DRG explants were overlaid with SFM and incubated at 37 °C until the time of harvest of inoculums (12, 24, and 36 h). Inoculum of DRG explants infected with HSV-1 or mock treated was laid on Vero cells cultured in triplicate to visualize plaque formation. In order to block secondary plaque formation, human immunoglobulin G (IgG; 1:10 Sigma-Aldrich, St. Louis, MO) was added to the inoculums. The Vero cells were washed with PBS buffer, fixed in ethanol 80%, and stained with Giemsa stain. Infectivity was quantified by counting the number of plaques.

### Immunofluorescence assay

DRG explants and SCNs were either uninfected or infected with HSV-1 (KOS-804) at a concentration of 1.0 multiplicity of infection (MOI) for SCNs and 10,000 virions for explants. Samples were incubated for 1 and 12 h in neurobasal media under 5% CO_2_ at 37 °C. After infection, they were fixed with 4% formaldehyde (Sigma-Aldrich, St. Louis, MO) for 1 h and washed with PBS three times for 15 min each. Explants were embedded in 3% agarose and sectioned at a thickness of 100 μm using a vibratome (Pelco 100). Both sections and SCNs were incubated in primary antibodies overnight at 4 °C. The following antibodies were used: anti-gD (Abcam, 1:10); anti-gB (Thermo Scientific, 1:1000); anti-TrkC (Abcam, 1:1000); anti-HS (US Biological, 1:200); anti-3-OST-2 (Santa Cruz, 1:500); and Phage display derived antibody HS4C3, reactive with 3-*O*S HS (Ten Dam et al. 2006), 1:10. Primary antibodies were diluted in PBS (0.3% Triton X, 0.1% sodium azide) and 1% normal serum (Vector laboratories; Burlingame, CA). The next day, DRG explants and SCNs were washed with PBS 3× for 15 min and incubated with secondary antibodies for 1 h for all conditions except the ones labeled with anti-3-*O*S HS HS4C3 antibody (Ten Dam et al. 2006), which were incubated for 2 h in secondary antibody (anti-VSV), in the dark at room temperature. The secondary antibodies used were Alexa Fluor 488 goat anti-mouse IgG (Life Technologies, Canada) at 1:500 dilution, Cy3-AffiniPure goat anti-rabbit IgG (H+L) (Li StarFish, Italy) at 1:500 dilution, donkey anti-rabbit (Invitrogen, Rockford, IL) at 1:400 dilution, donkey anti-goat (Invitrogen, Rockford, IL) at 1:400 dilution, and mouse monoclonal IgG to VSV-GVSV (P5D4, Santa Cruz, Dallas, TX) at 1:200 dilution. All other conditions that did not include anti-3-*O*S HS were washed with PBS 3× for 15 min. Samples labeled with anti-3-*O*S HS (HS4C3) antibody were further incubated with goat anti-mouse Alexa Fluor 488. The samples were finally mounted on a microscope slide using Vectashield Hard Set™ Mounting Medium with DAPI (Vector laboratories, Burlingame, CA). Samples were imaged using the Nikon Eclipse Ti A1R confocal microscope and analyzed using the NIS-Elements software.

### Heparanase assay

SCNs were pre-treated with heparanase I and II (Sigma-Aldrich, St. Louis, MO) at 1 unit/ml for 3 h at 37 °C with 5% CO_2_ (Tiwari et al. 2004). After pre-treatment of SCNs with heparanase and or mock treatment, the SCNs were infected with HSV-1 gL86 at 1 MOI for 2 h in neurobasal media at 37 °C with 5% CO_2_. The cells were then washed to remove any unbound viruses and were let to incubate in fresh neurobasal media overnight at 37 °C with 5% CO_2_ followed by the addition of *ortho*-nitrophenyl-β-d-galactopyranoside (ONPG) substrate (Pierce, Rockford, IL). The β-galactosidase activity was measured at 410 nm wavelength using the microplate reader (PerkinElmer EnSpire: multimode plate reader).

### Cytokine assay

Using a RayBio cytokine antibody array (RayBio, Norcross, GA), we assessed the expression of inflammatory markers during HSV-1 infection in mouse-derived DRG explants ex vivo (Baldwin et al. 2013). We used HSV-1 (KOS) 804 virus strain at a low concentration (10,000 virions) to infect DRG. DRGs mock infected with PBS were used as control. The supernatant of treated explants was collected after 12, 24, and 36 h post-infection. The cytokine antibody array was performed in accordance with the manufacturer’s instructions as previously described (Baldwin et al. 2013). The expression of each cytokine was evaluated in triplicate, quantified, and normalized as per manufacturer’s instructions (Fig. 4S).

## Results

### Susceptibility of mouse-derived DRG to HSV infection

We first isolated DRGs from adult NIH/Swiss mice and established an ex vivo model to study HSV-1 infection. A group of three DRG explants plated per well in triplicate experiment was challenged in a dosage-dependent manner with reporter β-galactosidase expressing HSV-1 KOS (gL86) virions at 37 °C or mock infected using the same culture conditions at various time points. Viral entry assay was quantified based on the amount of β-galactosidase expressed from the viral genome in which β-galactosidase expression is inducible by HSV infection (data not shown). Next, we determined HSV-1 replication by visualizing viral plaque assay (Fig. 2S). DRG explants were infected with replication-competent HSV-1 (KOS-804) strain (10,000 virions) or mock-infected explants at 37 °C for 24 h. To visualize plaque formation, Vero cells were overlaid with either the infected or the mock-infected DRG’s inoculum for 12, 24, and 36 h. The results show a larger number of plaques in a time-dependent manner. In contrast, no plaque was noticed in mock-infected DRGs (Fig. 2S).

Altogether, these results indicate the susceptibility of mouse-derived DRG ex vivo explants to HSV-1 infection.

### Expression of HS and localization of HSV-1 gB with HS in HSV-1-infected DRGs and SCNs

We next investigated the initial stages of HSV-1 infection in DRG and SCN models. Our focus was on HS, a widely recognized receptor involved in mediating HSV-1 attachment or binding at the host cell surface (Herold et al. 1991; Shieh et al. 1992). First, we confirmed the expression of HS in high-resolution confocal microscopy on both whole DRG tissue sections and DRG-derived SCN models (Fig. 2a, b). Immunofluorescence confocal microscopy confirmed an evenly distributed expression of HS (anti-HS antibody 10E4 epitope, US Biological) among the neuronal population in the whole DRG tissue section (Fig. 2a). Similarly, a wide expression of HS was also observed in DRG-derived SCN (Fig. 2b). The isolation of primary single cell DRG neurons does not account for purification of just neuronal cells. A sub-population of satellite cells is still present. Therefore, we used neuronal specific marker TrkC to confirm the neuronal phenotype of the cell population used for this study (Fig. 2a, b).

After confirming HS expression in both models, we then infected DRGs (10,000 virions) and SCNs (1.0 MOI), with HSV-1 (KOS) 804 virus for 1 h at 37 °C to visualize HSV-1 binding or attachment. As shown in Fig. 2c, d, a co-localization between HSV-1 gB and HS was clearly observed at the surface of the neuronal cell bodies in both models indicating the initial participation of HS in the event of viral attachment.

### Co-localization between HSV-1 gD and 3-*O*S HS in both DRGs and SCNs

**Fig. 3:**
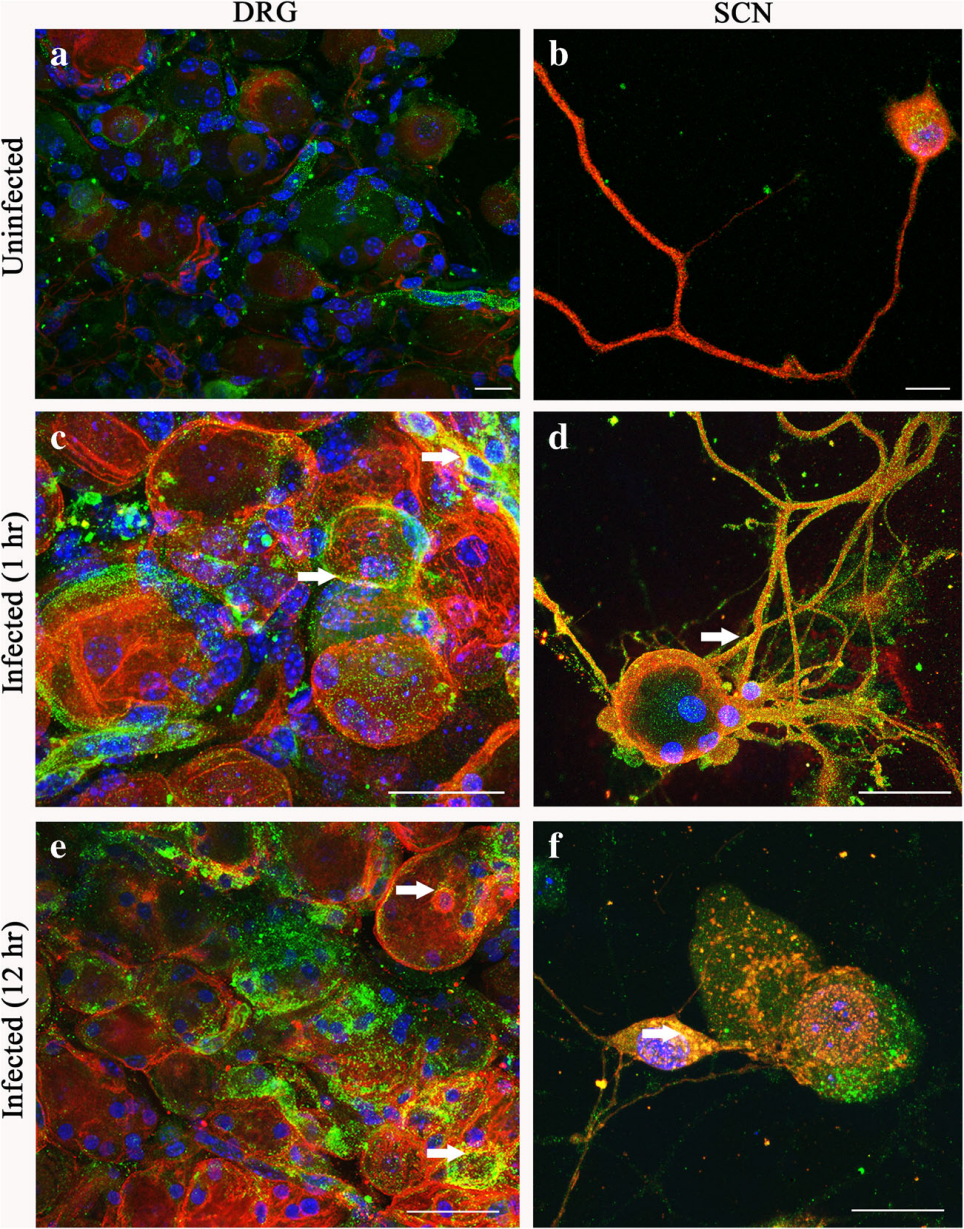
Expression and localization of 3-*O*-sulfated heparan sulfate (3-*O*S HS) in dorsal root ganglia (DRG) and single cell neurons (SCN). Sections of DRGs (**a**) and SCNs (**b**) were double labeled for anti-3-*O*S HS sulfate using the anti-HS (HS4C3) FITC-conjugated antibody and the specific neuronal marker against TrkC receptor (*red*) showing that 3-*O*S HS sulfate is widely expressed on the surfaces of the neuronal bodies. Samples were mounted on coverslips with mounting medium containing the nuclear stain blue DAPI. Both DRG tissue sections and SCNs were then infected with HSV-1 for either 1 (**c**, **d**) or 12 h (**e**, **f**), respectively. Double immunofluorescence with specific antibody for both HSV-1 glycoprotein gD (*red*) and 3-*O*S HS sulfate (*green*) revealed co-localization (*yellow*, *arrow*) of the two markers more in the neuronal cell surface at 1 h time point (**c**, **d**). In contrast, at 12 h, the co-localization of the two markers (*arrows*) is seen more internally, in the proximity of the nuclear region (**e**, **f**). The images were taken in confocal microscopy. *Scale bars*: **a**–**f** = 25 μm (color figure online)

We next evaluated if DRGs and SCNs express the modified version of HS which is 3-*O*-sulfated heparan sulfate (3-*O*S HS). The latter form of HS is a product of enzymatic modification by 3-*O*-sulfotransferase (3-*O*STs) (Esko and Lindahl 2001). Previous studies have shown that 3-*O*S HS, generated by 3-*O*ST-3 isoforms, allows HSV-1 entry by interacting to HSV-1 glycoprotein D (Shukla et al. 1999). The presence of 3-*O*ST-2 enzyme was confirmed in our neuronal models by immunofluorescence (Supplementary Fig. 3S). As indicated in Fig. 3a, b, a punctate labeling for -3-*O*S HS was observed on the cell surface of TrkC-positive neurons. This result provided the rationale to further examine the interaction between HSV-1 glycoprotein D (gD) and 3-*O*S HS, a step essential for virus-cell fusion (Shukla et al. 1999; Tiwari et al. 2004, 2007). These experiments were conducted using whole DRGs and SCNs infected with HSV-1 or mock infected for either 1 (Fig. 3c, d) or 12 h (Fig. 3e, f), respectively. Samples were fixed and immunolabeled with anti-HSV-1 gD and anti-3-*O*S HS (HS4C3) antibodies. As shown in Fig. 3c, d, HSV-1 gD-3-*O*S HS yellow co-localization at 1-h post-infection (p.i.) was observed at the surface of DRGs and SCNs, respectively. Interestingly, at later time point (12 h, p.i.), a similar yellow co-localization was observed in the mid-section of the sliced cells in both of the models (DRGs: Fig. 3e, and SCNs: Fig. 3f). The later results suggest the role of 3-*O*S HS receptor in HSV-1 trafficking.

### Enzymatic removal of cell surface HS significantly reduces HSV-1 entry in SCNs

**Fig. 4:**
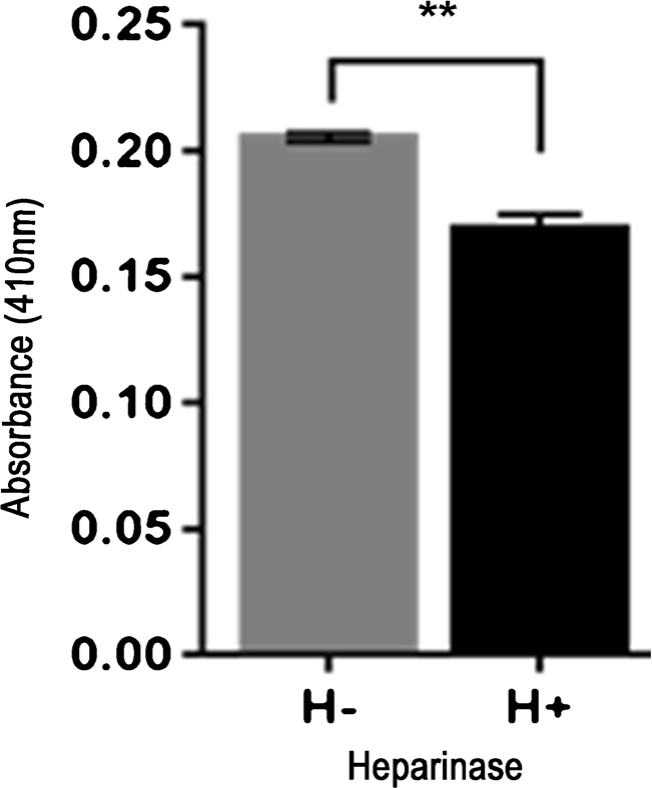
Enzymatic removal of cell surface HS results significant inhibition in HSV-1 entry in DRG-derived SCNs. SCNs were treated with heparanase I and II (1 U/ml; H^+^) or mock treated with 1× PBS (H^−^ for 3 h at 37 °C. SCNs were then challenged with β-galactosidase expressing reporter HSV-1 gL86 virus (1 MOI). After 12 h, cells were permeabilized and incubated with ONPG substrate for quantitation of β-galactosidase activity expressed from the input viral genome. Enzymatic activity was measured (H^+^ sample: 0.1691 ± 0.006; H^−^ sample: 0.2053 ± 0.002) by determining OD_410_. Data represent the mean ± the standard deviation of results in triplicate wells in a representative experiment. The experiment was repeated three times with similar results

To assess whether HS and 3-*O*S HS play a crucial role in virus entry, SCNs were pre-treated with enzymes heparanase I and II (1 U/ml). This enzyme selectively cleaves both heparin and HS chains containing 1 → 4 linkages between glucosamines and *O*-sulfated iduronic acid residues (Ernst et al. 1995). Mock-treated SCNs were used as control. Thereafter, SCNs were infected with β-galactosidase expressing HSV-1 gL86 reporter virions (1 MOI) for 2 h at 37 °C and 5% CO_2_. Quantification of HSV Lac *Z* expression showed a significant (*P* = 0.0036) decrease of viral entry in the heparanase treated SCNs (0.1691 ± 0.006) compared to mock-treated (0.2053 ± 0.002) control (Fig. 4). The previous results suggest a possible role for HS and 3-*O*S HS in HSV-1 entry in our single neuron model.

### Cytokine array analysis during HSV-1 infection in DRG explant

**Fig. 5:**
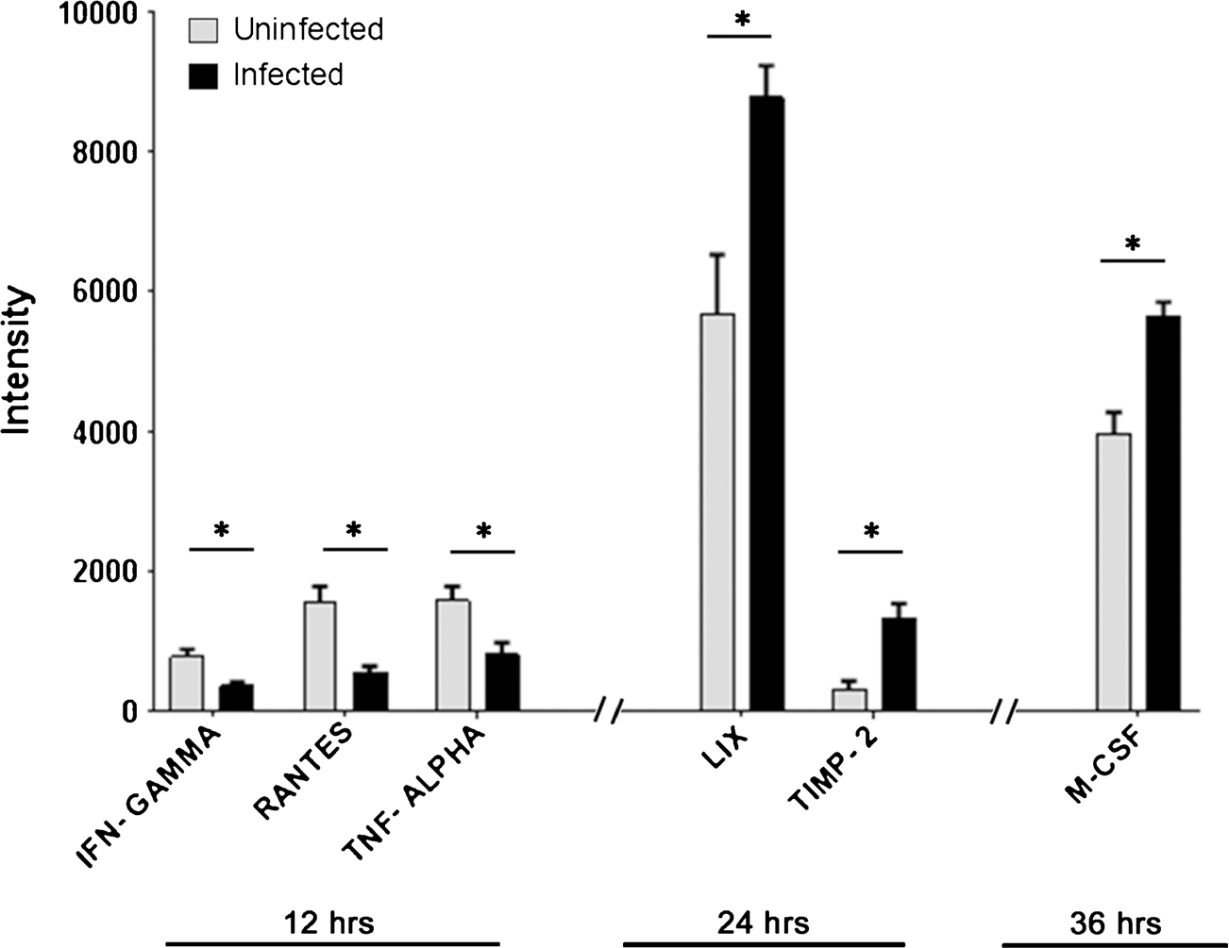
Inflammatory signals in DRG explant upon HSV-1 infection. A RayBio cytokine antibody array (RayBio, Norcross, GA) as per the manufacturer’s instructions was used to assess inflammatory markers produced in HSV-1 (KOS) 804 (10,000 virions) infection of ex vivo DRG explant at 12, 24, and 36 h. The experiment was run in triplicates. Signal intensities of various inflammatory markers are plotted in uninfected (*grey bars*) versus HSV-1 infected DRGs (*black bars*) at different time points. Inflammatory markers that resulted in significant decrease (IFN-γ (*P* = 0.027), RANTES (*P* = 0.011), and TNF-α (*P* = 0.038)) and increase (LIX (*P* = 0.032), TIMP-2 (*P* = 0.013), and M-CSF (*P* = 0.011)) in their signal intensities compared to their uninfected controls are indicated

Cytokines are a very heterogeneous family of molecules involved in inflammatory responses (Cavaillon and Haeffner-Cavaillon 1993; Newton and Dixit 2012; Mogensen 2009). Therefore, we sought to determine if DRG explants could produce inflammatory response to HSV-1 infection. A panel of more than 40 cytokines was tested in triplicate experiment in DRG explants infected with HSV-1 (KOS-804, 10,000 virions) at 12, 24, and 36 h p.i. Mock-infected DRGs were used as a control. The results of the entire panel are shown in the supplementary Fig. 4S. Interestingly, HSV-1 notably affected the expression of many members of the cytokine family at different time points. For instance, a quantitative analysis revealed a significant decrease in expression of interferon (IFN)-γ, RANTES, and TNF-α at 12 h p.i. On the contrary, other two members of the family, LIX and TIMP-2, significantly increase at 24 h. A similar result is seen for M-CSF at 36 h (Fig. 5).

## Discussion

In this study, we developed mouse-derived DRG explants and SCN models to investigate early stages of HSV-1 infectivity. Using confocal microscopy, we first demonstrated that diverse chains of HS (HS and 3-*O*S HS) are present on the surface of DRGs and SCNs as evident from the results of the immunofluorescence experiments (Figs. 2 and 3). The variability in the HS chain has been associated with the generation of binding sites for specific ligands and therefore plays a critical role in maintaining and performing multiple biological functions including neuronal growth (Esko and Lindahl 2001; Mogensen and Paludan 2001; Thacker et al. 2016; Bishop et al. 2007). On the other hand, multiple pathogens including herpes viruses exploit variability in HS chain in their benefit by facilitating pathogenesis (Tiwari et al. 2012; O’Donnell and Shukla 2008). Our result established the first step of HSV-1 attachment/binding by demonstrating the co-localization between glycoprotein B (gB) to HS (Fig. 2). In addition, we also provided evidence of virus-cell fusion by co-localization between HSV-1 gD and 3-*O*S HS (Fig. 3). We further tested if HS and 3-OS HS were critical for HSV-1 entry in our model systems. Upon enzymatic treatment with heparanase I and II, which selectively cleaves sulfated residues in HS, a significant reduction in HSV infection was noticed in the DRG-derived SCNs suggesting the significance for HS/3-*O*S HS in mediating virus infection (Fig. 4).

Finally, to gain more detail insight to the post-infection inflammatory response in DRG model, a previously described chemokine array analysis was performed (Baldwin et al. 2013). We noticed significant increase (LIX, TIMP-2, and M-CSF) and decrease (IFN-γ, RANTES, and TNF-α) in specific chemokines, which are interestingly known to influence inflammatory response either by binding directly to HS and sulfated HS or by affecting HS-dependent or HS-independent signaling pathways (Lortat-Jacob et al. 2002; Lortat-Jacob 2009; Davis and Parish 2013; Wang and Knaut 2014). Several interesting observations were generated from our chemokine array analysis. For instance, we noticed an enhanced expression of neurospecific TIMP-2 in HSV-1-infected cell. Interestingly, an upregulation of TIMP-2 has been linked to FGF/EGF signaling that controls cell proliferation, differentiation, neurite growth (Fager and Jaworski 2000; Young et al. 2002), and markers for the ECM accumulation and fibrosis (Jaworski and Pérez-Martínez 2006; Arpino et al. 2015). The increase observed for M-CSF in our model of HSV-1 infection correlates with data previously obtained in P19 neuronal-like cells infected with HSV-1 (Smith et al. 2013; Dixit et al. 2008). In that case, as well as for other models, an enhancement of M-CSF in the brain was linked to cytoskeleton reorganization, phagocytosis, and several disease processes including neuroinflammation (Smith et al. 2013; Dixit et al. 2008). In addition, our previous finding showed a novel phagocytic uptake of HSV-1 as a primary mechanism in corneal fibroblasts derived from human eye donors (Clement et al. 2006). Further investigation will determine if M-CSF plays a major role in phagocytic uptake of HSV-1.

Similarly, an enhanced signal for LIX (CXCL5) was observed in HSV-1-infected DRG explants compared to mock infected (Fig. 5). LIX is a well-known marker for neuroinflammation, and it has been reported to be upregulated in response via a phosphatidylinositol 3-kinase (PI3 kinase) and NF-kappa B pathway. As previously reported, PI3 kinase signaling affects multiple steps during HSV entry (Tiwari and Shukla 2010).

Finally, a significant reduction of RANTES (CCL5), TNF-α, and IFN-γ was observed during early phase of HSV-1 infection (Fig. 5). Interestingly, RANTES is a key player in enhancing viral binding or attachment by cross-linking virus and cell, a process that involves chemokine-HS binding (Trkola et al. 1999). The expression of TNF-α and IFN-γ neuroinflammatory markers increases at 24–36 h (Fig. 4S) post-HSV-1 infection (Olmos and Lladó 2014).

In summary, with this study, we established ex vivo DRG explants and DRG-derived SCNs as model systems to study HSV-1 entry and associated neuroinflammation. Our data clearly suggest the involvement of HS and 3-*O*S HS in mediating HSV-1 infection. Further investigations are required to address which inflammatory cytokine are specifically induced under which sub-set of sulfated HS (Lawrence et al. 2007). These findings, together with additional information on the critical epitopes involved during HSV-1 gD interaction with 3-*O*S HS, will advance our current understanding on the HSV-1-induced pathogenesis, neuroinflammation, and tissue damage. The results of these experiments will be extremely useful for the development of the novel therapeutic interventions.

## Electronic supplementary material

1S **Viral entry and β-galactosidase expression.** DRG explants infected with HSV-1 KOS (gL86) carrying β-galactosidase reporter gene forms blue cells as a result of viral entry. The β-galactosidase enzyme catalyzes the substrate (5-bromo-4-chloro-3-indolyl-β-D-galactopyranoside; X-gal) by hydrolysis. Higher magnifications of the stained cells within the boxed areas are magnified in the inset pictures (A-D). All figure's bar = 25μm. (GIF 541 kb)

High resolution image (TIFF 23696 kb)

2S **HSV replication and spread in DRG explants using plaque assay.** DRG explants infected with replication competent HSV-1 (KOS 804 – 10,000 virions) strain (B, D, F) or mock infected explants (A, C, E) were laid on Vero cells for 12, 24 and 36 hours. Cells were fixed with 4% PFA and stained with Giemsa. An increase in cytopathic effect (plaque formation) was observed over time (D, F) in Vero cells indicating viral spread. (GIF 711 kb)

High resolution image (TIFF 29615 kb)

3S **Expression of 3-OST-2 enzyme.** 3-O-sulfotransferases 2 (3-OST-2) is an enzyme that adds a sulfate group to the 3-OH position of HS’s glucosamine residue. Immunofluorescence double-labelling revealed 3-OST-2 (green) expressed in the cytoplasm of TrkC-positive (red) neurons throughout the DRG explant (A) and in the DRG-dissociated single neurons (B) models. hoechst (blue) was used as nuclear marker. Scale bar = 25um. (GIF 111 kb)

High resolution image (TIFF 7192 kb)

4S **Cytokine array analysis.** Supernatant of DRG explants infected with HSV-1 (KOS-804) strain or mock infected were collected in triplicate after 12, 24 and 36 hours. The collected supernatant was used for a mouse inflammation antibody array (RayBiotech Inc) to detect the expression of forty cytokines. The three panels show the overall result of the pattern of expression of all forty cytokines at 12h (top panel), 24h (middle panel) and 36 h (bottom panel). The schematic representation is the result of an average quantification (and standard deviation) of the experiments in triplicate. (GIF 231 kb)

High resolution image (TIFF 22232 kb)
