# Supplementary figures and images for: HSV-1 interaction to 3-O-sulfated heparan sulfate in mouse-derived DRG explant and profiles of inflammatory markers during virus infection
Source: J Neurovirol. 2017 Mar 21;23(3):483–91. doi: 10.1007/s13365-017-0521-4 (PMC5440488; doi:10.1007/s13365-017-0521-4)

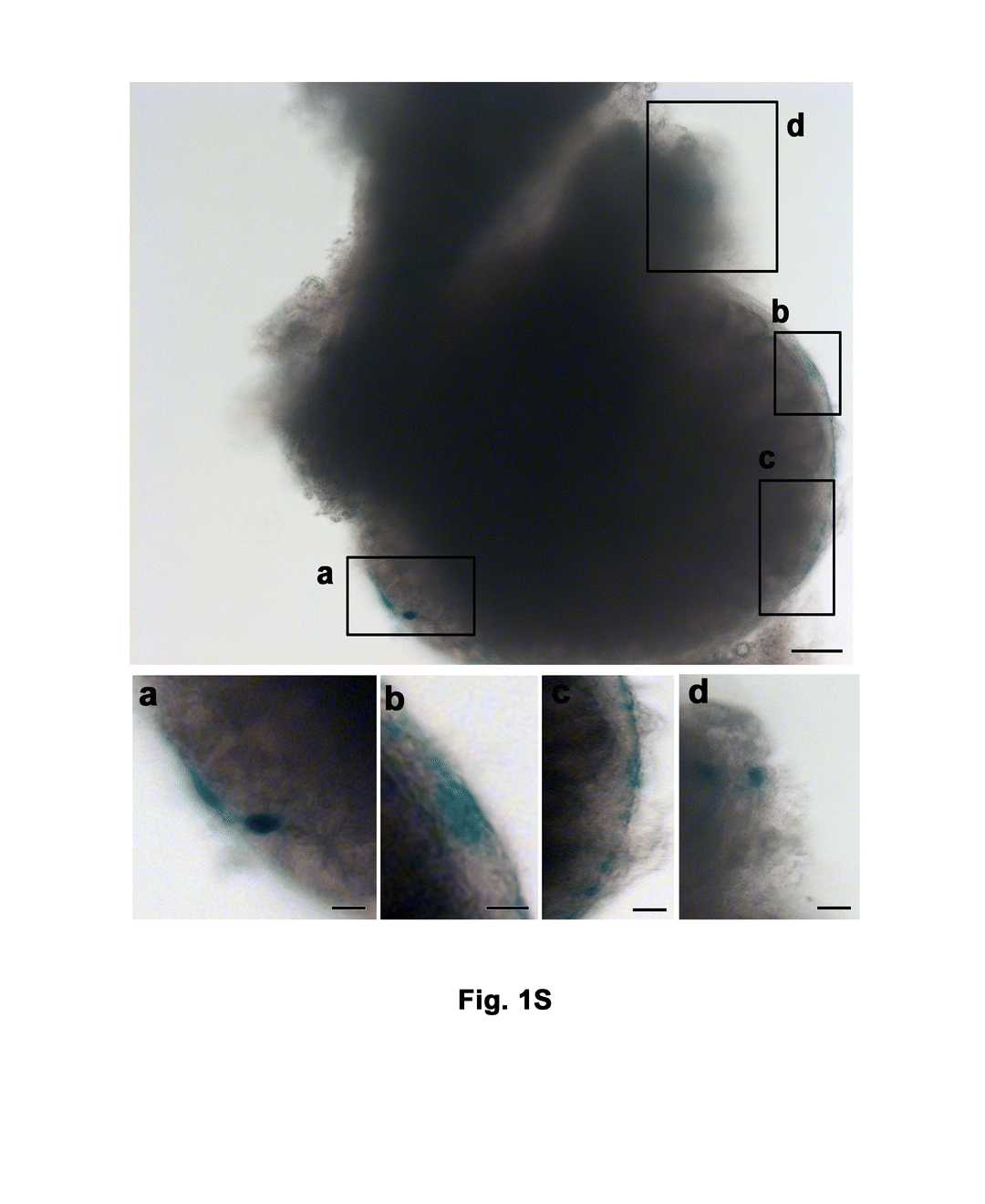

Supplement: Supplementary file 1 — Viral entry and β-galactosidase expression. DRG explants infected with HSV-1 KOS (gL86) carrying β-galactosidase reporter gene forms blue cells as a result of viral entry. The β-galactosidase enzyme catalyzes the substrate (5-bromo-4-chloro-3-indolyl-β-D-galactopyranoside; X-gal) by hydrolysis. Higher magnifications of the stained cells within the boxed areas are magnified in the inset pictures (A-D). All figure's bar = 25μm. (GIF 541 kb) [file 13365_2017_521_Fig6_ESM.gif]

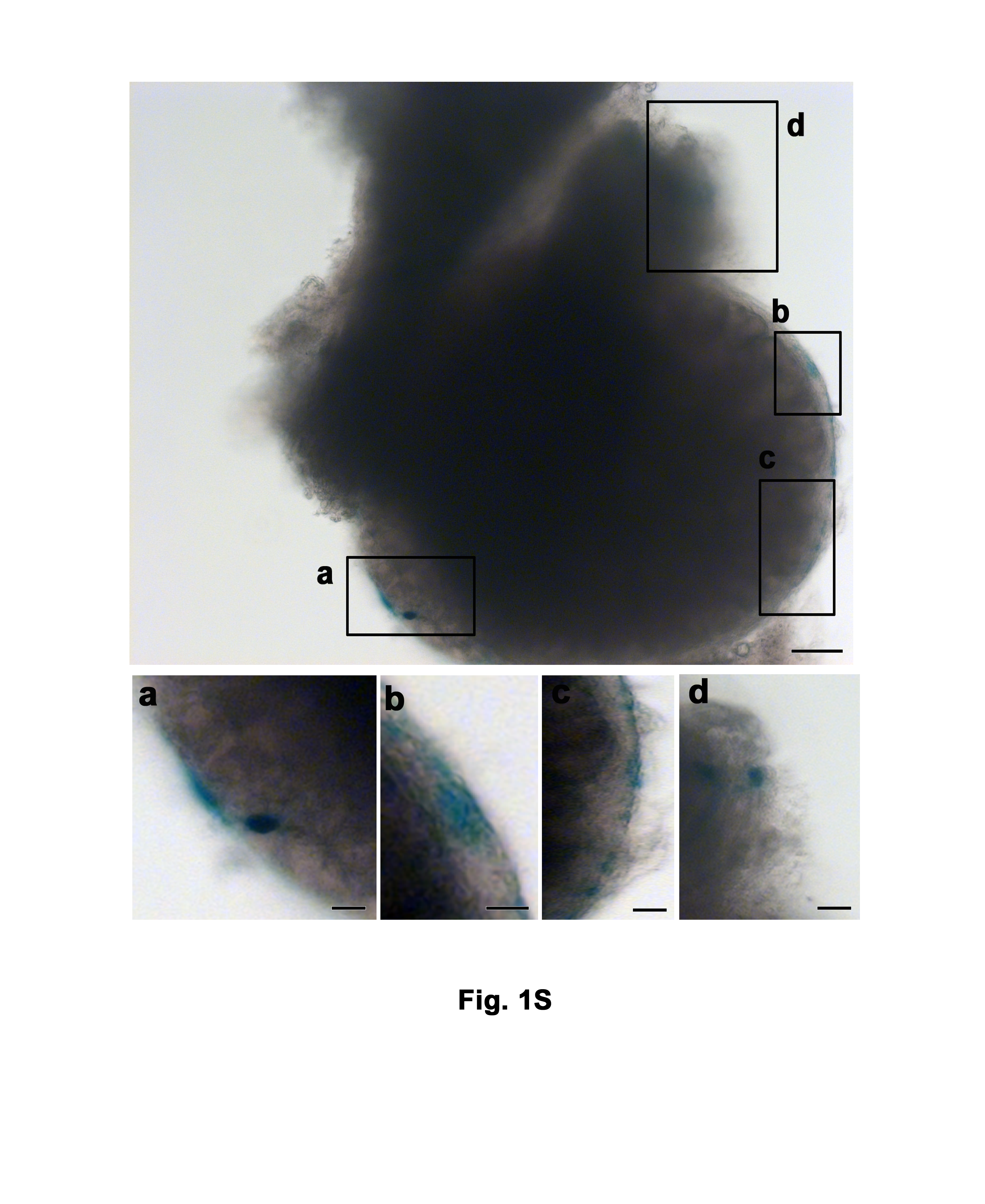

Supplement: Supplementary file 2 — High resolution image (TIFF 23696 kb) [file 13365_2017_521_MOESM1_ESM.tif]

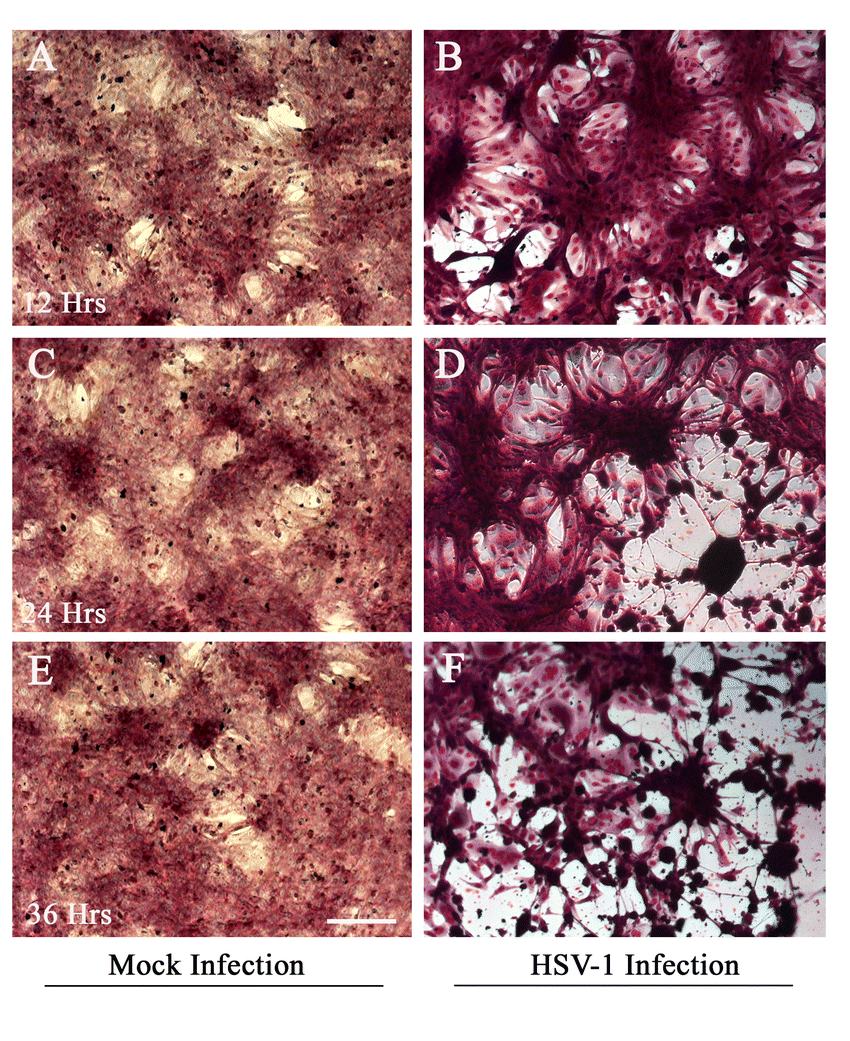

Supplement: Supplementary file 3 — HSV replication and spread in DRG explants using plaque assay. DRG explants infected with replication competent HSV-1 (KOS 804 – 10,000 virions) strain (B, D, F) or mock infected explants (A, C, E) were laid on Vero cells for 12, 24 and 36 hours. Cells were fixed with 4% PFA and stained with Giemsa. An increase in cytopathic effect (plaque formation) was observed over time (D, F) in Vero cells indicating viral spread. (GIF 711 kb) [file 13365_2017_521_Fig7_ESM.gif]

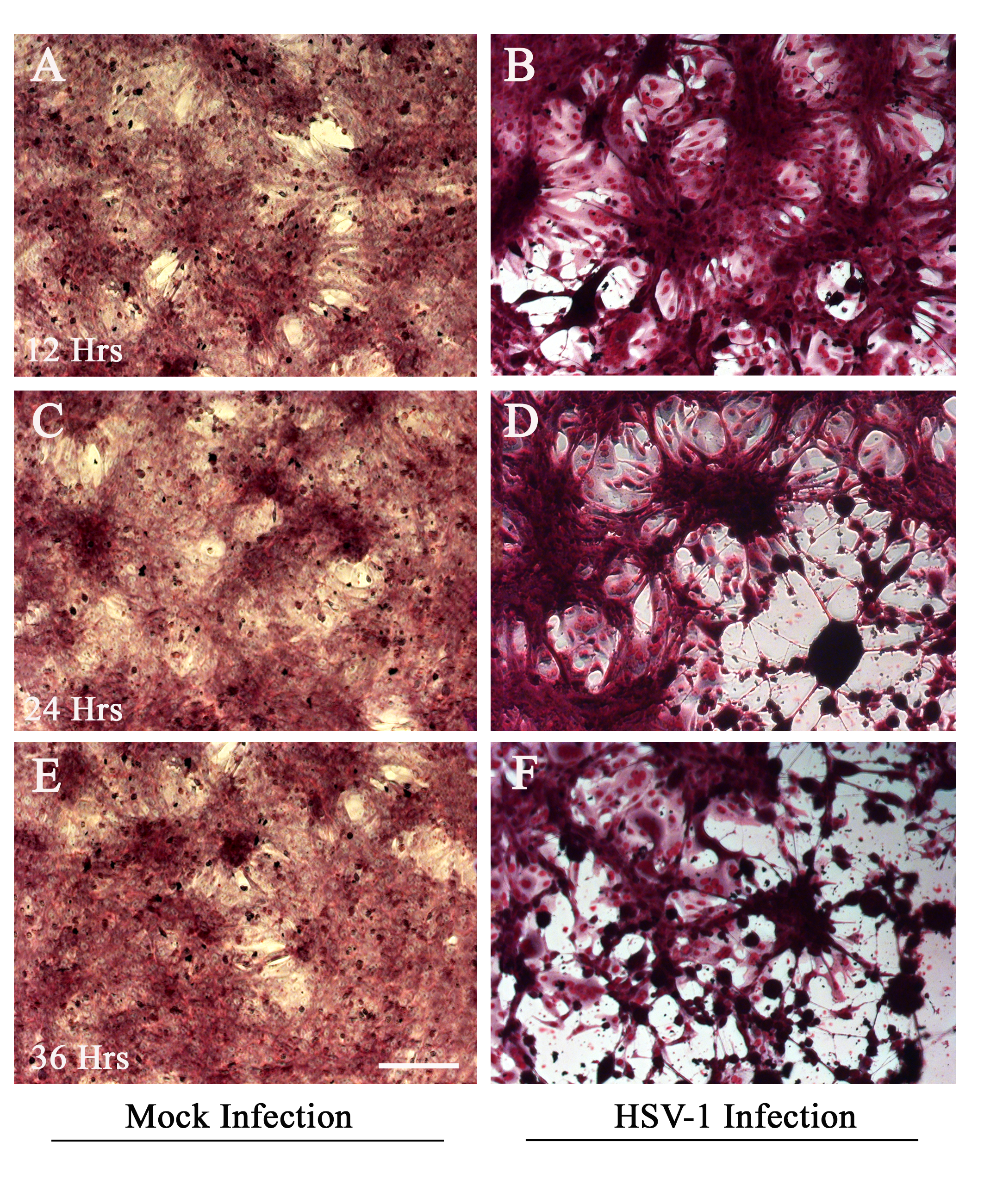

Supplement: Supplementary file 4 — High resolution image (TIFF 29615 kb) [file 13365_2017_521_MOESM2_ESM.tif]

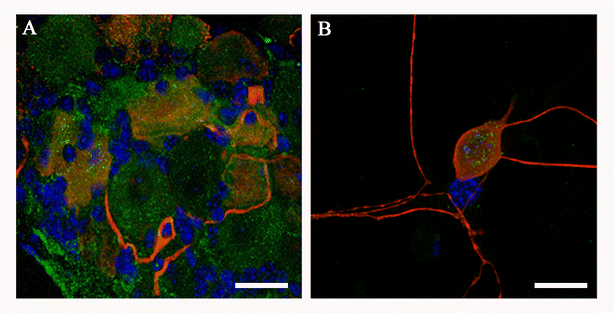

Supplement: Supplementary file 5 — Expression of 3-OST-2 enzyme. 3-O-sulfotransferases 2 (3-OST-2) is an enzyme that adds a sulfate group to the 3-OH position of HS’s glucosamine residue. Immunofluorescence double-labelling revealed 3-OST-2 (green) expressed in the cytoplasm of TrkC-positive (red) neurons throughout the DRG explant (A) and in the DRG-dissociated single neurons (B) models. hoechst (blue) was used as nuclear marker. Scale bar = 25um. (GIF 111 kb) [file 13365_2017_521_Fig8_ESM.gif]

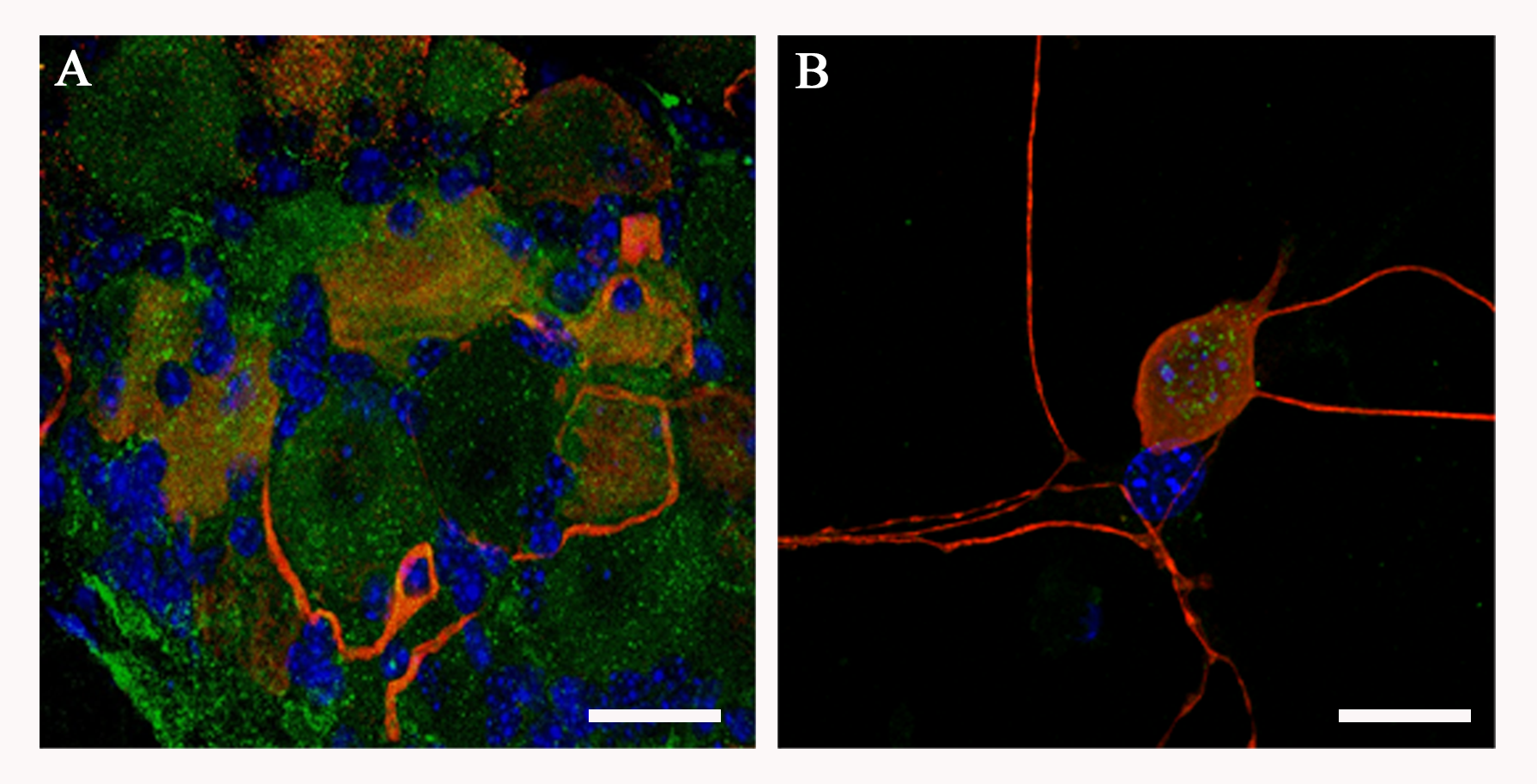

Supplement: Supplementary file 6 — High resolution image (TIFF 7192 kb) [file 13365_2017_521_MOESM3_ESM.tif]

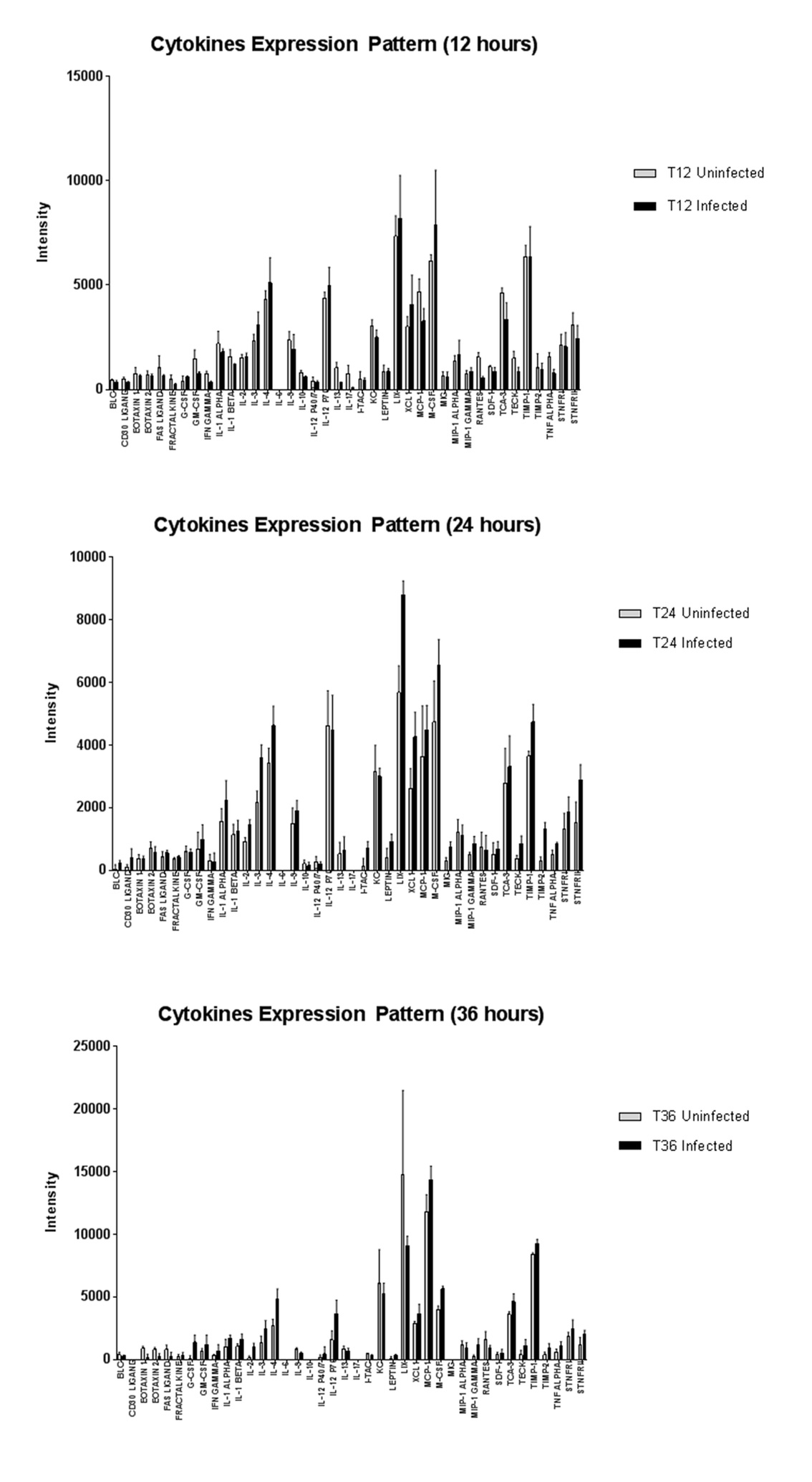

Supplement: Supplementary file 7 — Cytokine array analysis. Supernatant of DRG explants infected with HSV-1 (KOS-804) strain or mock infected were collected in triplicate after 12, 24 and 36 hours. The collected supernatant was used for a mouse inflammation antibody array (RayBiotech Inc) to detect the expression of forty cytokines. The three panels show the overall result of the pattern of expression of all forty cytokines at 12h (top panel), 24h (middle panel) and 36 h (bottom panel). The schematic representation is the result of an average quantification (and standard deviation) of the experiments in triplicate. (GIF 231 kb) [file 13365_2017_521_Fig9_ESM.gif]

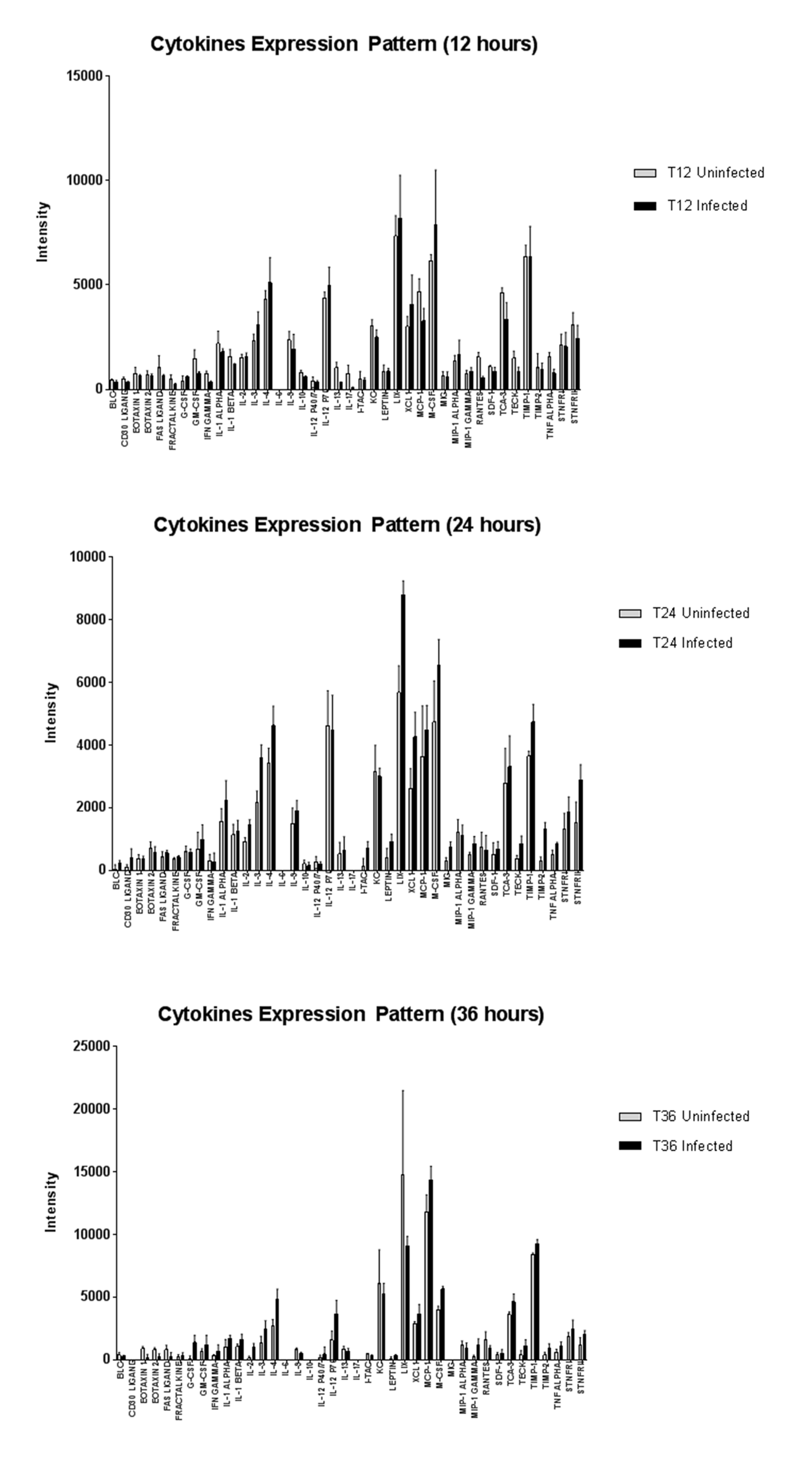

Supplement: Supplementary file 8 — High resolution image (TIFF 22232 kb) [file 13365_2017_521_MOESM4_ESM.tif]
